# Outcomes and prognostic factors in initially unresectable hepatocellular carcinoma treated using conversion therapy with lenvatinib and TACE plus PD-1 inhibitors

**DOI:** 10.3389/fonc.2023.1110689

**Published:** 2023-01-30

**Authors:** Xingzhi Li, Jie Chen, Xiaobo Wang, Tao Bai, Shaolong Lu, Tao Wei, Zhihong Tang, Chengwen Huang, Bin Zhang, Bowen Liu, Lequn Li, Feixiang Wu

**Affiliations:** ^1^ Department of Hepatobiliary Surgery, Guangxi Medical University Cancer Hospital, Nanning, China; ^2^ Key Laboratory of High-Incidence-Tumor Prevention & Treatment, Ministry of Education, Nanning, China

**Keywords:** conversion therapy, PD-1 inhibitors, initially unresectable hepatocellular carcinoma, transcatheter arterial chemoembolization, lenvatinib

## Abstract

**Purpose:**

To evaluate the outcomes and prognostic factors for patients using conversion therapy with lenvatinib combined with transcatheter arterial chemoembolization (TACE) plus programmed cell death protein-1 (PD-1) inhibitors (LTP) for initially unresectable hepatocellular carcinoma (iuHCC).

**Methods:**

Data on 94 consecutive patients with iuHCC who received LTP conversion therapy from November 2019 to September 2022 were retrospectively analyzed. Early tumor response was reported when patients showed complete or partial response at the time of their first follow-up (4–6 weeks) after initial treatment, in accordance with mRECIST. The endpoints consisted of conversion surgery rate, overall survival (OS), and progression-free survival (PFS).

**Results:**

Early tumor response was found in 68 patients (72.3%) and not in the remaining 26 patients (27.7%) in the entire cohort. Early responders had a significantly higher conversion surgery rate than non-early responders (44.1% vs. 7.7%, p=0.001). Early tumor response was the only factor independently associated with successful conversion resection, as indicated by multivariate analysis (OR=10.296; 95% CI: 2.076–51.063; p=0.004). Survival analysis showed that early responders had longer PFS (15.4 vs. 7.8 months, p=0.005) and OS (23.1 vs. 12.5 months, p=0.004) than non-early responders. Early responders who underwent conversion surgery also had significantly longer median PFS and OS (not reached, not reached) than those who did not (11.2 months, p=0.004; 19.4 months, p<0.001). In multivariate analyses, early tumor response was identified as an independent prognostic factor for longer OS (HR=0.404, 95% CI: 0.171–0.954; p=0.039). Successful conversion surgery was also an independent predictive factor for longer PFS (HR=0.248, 95% CI: 0.099–0.622; p=0.003) and OS (HR=0.147, 95% CI: 0.039–0.554; p=0.005).

**Conclusions:**

Early tumor response is an important predictive marker for successful conversion surgery and prolonged survival in patients with iuHCC treated using LTP conversion therapy. Conversion surgery is necessary to improve survival during conversion therapy, particularly for early responders.

## Introduction

Liver cancer is the fourth leading cause of cancer-related mortality (1). Surgical resection can provide a good prognosis for resectable hepatocellular carcinoma (HCC). However, many patients are ineligible for resection because of excessive tumor burden, large vascular invasion, intrahepatic metastases, or external metastases (2). Conversion therapy aims to provide an improved prognosis by converting unresectable hepatocellular carcinoma for a chance to receive curative surgery. In clinical practice, multidrug combination therapy has been explored in intermediate to advanced HCC (3–5). Lenvatinib combined with transcatheter arterial chemoembolization (TACE) plus programmed cell death protein-1 (PD-1) inhibitors (LTP) has shown improved prognosis in initially unresectable hepatocellular carcinoma(iuHCC) (5–7). The objective response rate of LTP are higher than those of TACE alone and TACE combined with lenvatinib (6, 7). Thus, triple-combination therapy can potentially serve as a conversion therapy.

Achieving tumor shrinkage downstaging is an important goal in conversion therapy for the treatment of iuHCC. It provides patients with the opportunity for potential radical resection (2, 8). About 50% of patients have been found to exhibit obvious tumor shrinkage after triple-combination therapy and offered curative conversion resection (9, 10). In this context, early prediction of successful conversion surgery may guide surgical treatment strategies. Moreover, early identification of patients not benefitting from conversion therapy and timely switching to other second-line treatments may improve the prognosis.

Early tumor response could respond to the reduction of tumor burden from radiological evaluation earlier. It has been associated with good prognosis in numerous malignancies (11–17). In HCC patients receiving sorafenib, lenvatinib, and combined therapy, early tumor shrinkage can lead to improved outcomes and extended survival (18–20). However, early tumor response based on the modified Response Evaluation Criteria in Solid Tumors (mRECIST) does not prolong survival in HCC patients receiving PD-1 inhibitors plus bevacizumab (21). Thus, although early tumor response represents a rapid reduction in tumor burden, whether it has a predictive role in patients treated with immune combination therapy, particularly LTP conversion therapy, remains inconclusive.

This retrospective study was aimed at evaluating outcomes and prognostic factors for patients who underwent LTP conversion therapy for iuHCC.

## Methods

### Study population

Consecutive patients who received LTP conversion therapy from November 2019 to September 2022 for iuHCC were retrospectively analyzed. HCC was diagnosed by clinical assessment or histological examination (22). Tumors were considered unresectable either because of technical unresectability or oncologic unresectability, or both. Technical unresectability is defined in the presence of insufficient future remnant liver volume and lesions assessed by the surgeon as unsuitable for R0 resection. Oncological unresectability is defined as failure to obtain a better prognosis after surgical resection of intermediate-to-advanced HCC, such as major vascular invasion, intrahepatic metastases, and extrahepatic metastases. Other inclusion criteria were as follows: age between 18 and 80 years; Eastern Tumor Collaborative Group physical status (ECOG-PS) score of 0 to 1; Child–Pugh class A or B; adequate organ function; the absence of previous systemic and local treatment history for HCC, and at least one measurable target lesion in accordance with mRECIST. Those who received no radiologic evaluation at the first follow-up 4–6 weeks after the initial treatment for any reason were excluded. The ethics committee at our institution examined and approved the study protocol (LW2022147), and written informed consent was obtained from each patient.

### Treatment strategies

Superselective TACE was performed by specialists with extensive surgical experience in the interventional unit (details in supplementary material). Repeat TACE was conducted when the active lesion area exceeded 50% of the baseline while the liver function was reasonable. Lenvatinib (LENVIMA^®^, Merck Sharp & Dohme, H20180052) and PD-1 inhibitors were given within 1 week after TACE, depending on the general condition and recovery of liver function. Patients were given either 8 mg (weight <60 kg) or 12 mg (weight ≥60 kg) of lenvatinib daily *via* the oral route. PD-1 inhibitors included either 200 mg of camrelizumab (AiRuiKa^®^, Jiangsu Hengrui Medicine Co. Ltd, S20190027) or 200 mg of sintilimab (Tyvyt^®^, Innovent Biologics and Eli Lilly and Company, S20180016) *via* intravenous injection every 3 weeks. All patients were treated until disease progression, intolerable toxicity, death, or withdrawal for any reason.

Hepatectomy was performed if patients met the criteria for resection and informed consent was obtained. The criteria for tumor resectability had to be satisfied, as follows: (1) adequate residual liver volume; (2) adequate cutting edge to achieve R0 resection; (3) tumor response assessment of complete response (CR) or partial response (PR) in accordance with mRECIST criteria, or patients with efficacy assessment of stable disease (SD) and maintenance for more than 8 weeks were considered to have controlled tumor biological behavior; (4) Child–Pugh grade A or B, and no other contraindications to hepatectomy.

### Follow-up

The first follow-up was scheduled 4–6 weeks after the initial treatment and then every 2–4 months until October 20, 2022. Each follow-up evaluation includes tumor response and laboratory tests, such as blood tests, liver and kidney function, urine routine, tumor markers, myocardial enzymology examination, and thyroid function. Tumor response was evaluated on contrast-enhanced computed tomography (CE-CT) using mRECIST and RECIST1.1 by two senior hepatobiliary surgeons at our hospital.

### Outcome assessments

Early tumor response was defined as patients with CR or PR at the time of the first follow-up (4–6 weeks) after initial treatment using mRECIST, whereas late tumor response was documented after the first follow-up. The non-early response included late tumor response, SD, or progressive disease (PD). Early α-fetoprotein (AFP) response was defined as a reduction of more than 75% in AFP levels following the initial treatment at the first follow-up (23).

We also evaluated the prognostic factors by using inflammatory indexes such as NLR, PLR, and systemic inflammation response index (SIRI). SIRI was calculated as neutrophil count × monocyte count/lymphocyte count (24). These inflammatory indices were split into two groups, based on their median value. Overall survival (OS) was measured from the initiation of the conversion therapy to death from any cause. Progression-free survival (PFS) was calculated from the initiation of the conversion therapy to progression, relapse, or death.

### Statistical analysis

The data collected in this study were statistically analyzed using the software SPSS ver. 24.0 (IBM, Armonk, NY, United States) and R ver. 4.1.1 (http://www.R-project.org/). The Mann–Whitney U test or t-test was used to compare continuous variables, represented as median and quartiles or mean ± standard deviation. Categorical variables were presented as the number of cases and percentages by using the χ² test or Fisher’s exact probability method. Survival analysis was conducted using Kaplan–Meier methods and log-rank tests. The inverse Kaplan–Meier method was conducted to determine the median time of follow-up.

Potential predictive factors of successful conversion surgery were determined using binary logistic regression methods. In multivariate analysis, all factors with p<0.05 and clinically important variables in the univariate analyses were included *via* the enter method. Given the clinical correlation between early tumor response and AFP response, two models were used to include early tumor response and AFP response in separate multivariate logistic regression analyses to avoid collinearity. Considering that no patients with distant metastases had successful conversion surgery, we presented a sensitivity binary logistic regression analysis for patients without distant metastases.

Potential prognostic factors for PFS and OS were determined using the Cox proportional-hazards models. All factors with p<0.05 and clinically important variables for prognosis were included in the multivariate analysis *via* the enter method. Given the correlation between successful conversion surgery and early tumor response, we included two models in separate multivariate cox regression analyses to avoid collinearity. For all analyses, p<0.05 was considered statistically significant.

## Results

### Patient characteristics

**Figure 1:**
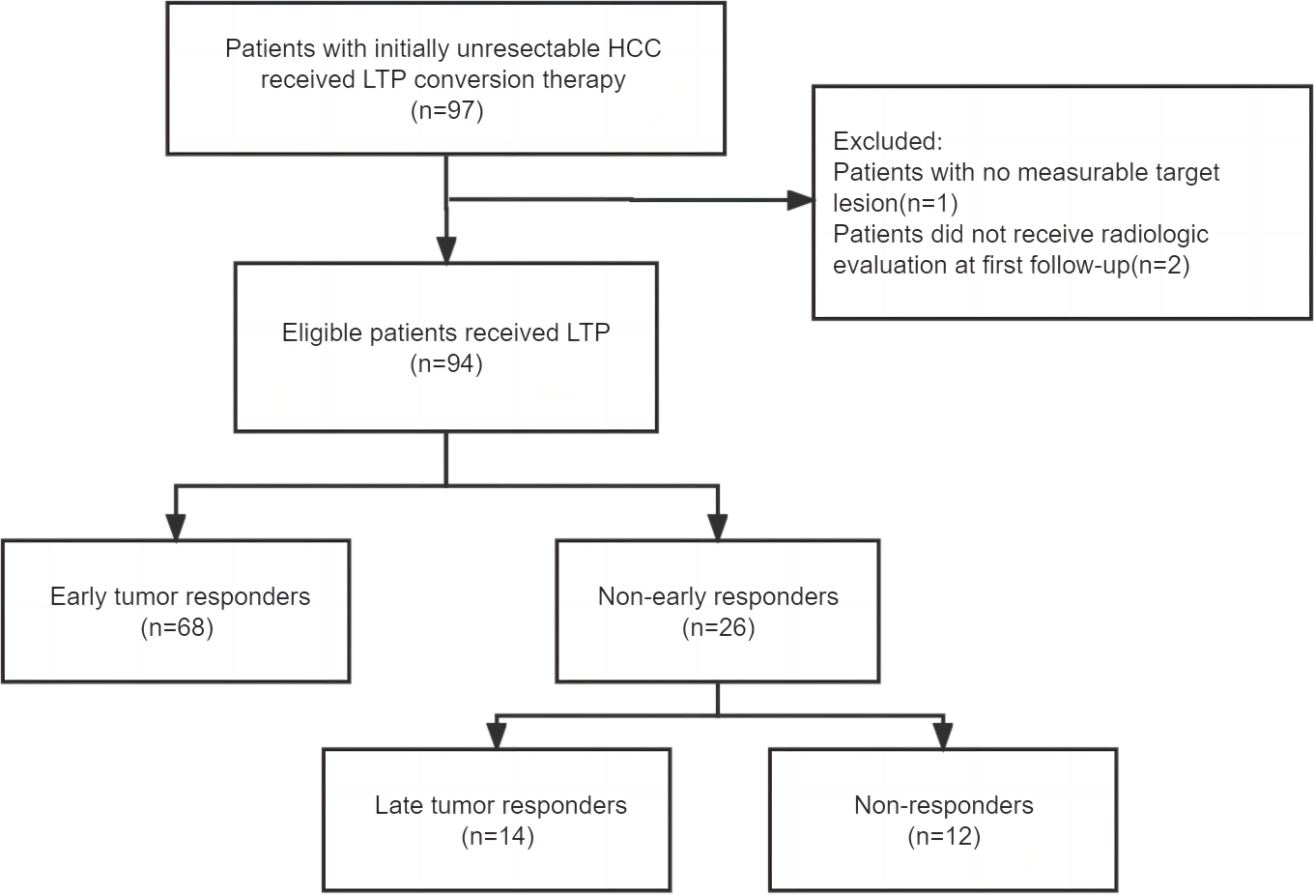
Flowchart. LTP, Lenvatinib combined TACE plus PD-1inhibitors.

A total of 97 patients who received conversion therapy to treat iuHCC were assessed; 3 patients were subsequently excluded (**Figure 1**). Among the 94 patients included, 84 (89.4%) had hepatitis B-associated HCC. At baseline, 85 patients (90.4%) were classified as Child–Pugh grade A, and the majority were assigned with an ALBI grade >1 (88.3%). Further, 60 (63.8%) were of Barcelona clinical liver cancer (BCLC) stage C and 19 cases (20.2%) were of BCLC stage B; 56 (59.6%) patients had an initial AFP > 400 ng/mL. In 70.2% of patients, the tumors measured >10 cm in diameter. Multiple tumors were found in 51 patients (54.3%). The laboratory tests were also summarized, including the results for the hepatobiliary enzyme, total bilirubin, NLR, PLR, and SIRI (**Table 1**). Although adverse events in varying degrees affected all patients, they were within controllable levels (**Table S1**).

**Table 1:** Baseline characteristics of 94 patients with initially unresectable HCC.

Variable	Early tumor responders (n=68)	Non-early responders (n=26)	P value
Age (year)			0.344
>60	9 (13.2%)	6 (23.1%)	
≤60	59 (86.8%)	20 (76.9)	
Gender			1.000
Male	64 (94.1%)	25 (96.2%)	
Female	4 (5.9%)	1 (3.8%)	
BCLC stage			0.254
A	12 (17.7%)	3 (11.5%)	
B	16 (23.5%)	3 (11.5%)	
C	40 (58.8%)	20 (77.0%)	
Target tumor size (cm)			0.212
>10	45 (66.2%)	21 (80.8%)	
≤ 10	23 (33.8%)	5 (19.2%)	
Tumor number			0.362
1	29 (42.6%)	14 (53.8%)	
≥2	39 (57.4%)	12 (46.2%)	
Large vascular invasion			0.192
Yes	37 (54.4%)	18 (69.2%)	
No	31 (45.6%)	8 (30.8%)	
PVTT			0.679
Yes	36 (52.9%)	15 (57.7%)	
No	32 (47.1%)	11 (42.3%)	
Extrahepatic metastases			0.066
Yes	5 (7.4%)	6 (23.1%)	
No	63 (92.6%)	20 (76.9%)	
AFP (ng/mL)			0.810
>400	40 (58.8%)	16 (61.5%)	
≤ 400	28 (41.2%)	10 (38.5%)	
Etiology			0.456
Hepatitis B	62 (91.2%)	22 (84.6%)	
Non−Hepatitis B	6 (8.8%)	4 (15.4%)	
ECOG PS			0.030
0	43 (63.2%)	10 (38.5%)	
1	25 (36.8%)	16 (61.5%)	
Cirrhosis			0.669
Yes	55 (80.9%)	20 (76.9%)	
No	13 (19.1%)	6 (23.1%)	
Ascites			1.000
Yes	10 (14.7%)	3 (11.5%)	
No	58 (85.3%)	23 (88.5%)	
Child-Pugh grade			0.704
A	62 (91.2%)	23 (88.5%)	
B	6 (8.8%)	3 (11.5%)	
ALBI grade			0.739
1	7 (10.3%)	4 (15.4%)	
2	59 (86.8%)	22 (84.6%)	
3	2 (2.9%)	0 (0.0%)	
TBIL (umol/L)	17.5 (11.6-22.4)	15.2 (12.9-18.6)	0.348
ALB (g/L)	37.3 ± 4.3	36.3 ± 4.7	0.310
PT (sec)	12.9 ± 1.6	12.7 ±1.1	0.703
ALT (U/L)	42.5 (31.0-58.5)	49.0 (35.2-75.5)	0.199
AST (U/L)	57.0 (43.5-89.0)	75.5 (61.8-97.8)	0.020
PLT (×10^9^/L)	207.5 (164.2-257.5)	216.0 (151.8-288.5)	0.577
NLR			1.000
>2.82	34 (50%)	13 (50%)	
≤2.82	34 (50%)	13 (50%)	
PLR			0.356
>146	32 (47.1%)	15 (57.7%)	
≤146	36 (52.9%)	11 (42.3%)	
SIRI			1.000
>1.38	34 (50%)	13 (50%)	
≤1.38	34 (50%)	13 (50%)	
successful conversion surgery			0.001
Yes	30 (44.1%)	2 (7.7%)	
No	38 (55.9%)	24 (92.3%)	

ALT, alanine aminotransferase; BCLC, Barcelona Clinic Liver Cancer; ALBI, albumin–bilirubin; HCC, hepatocellular carcinoma; AFP, α-fetoprotein; ECOG PS, Eastern Cooperative Oncology Group performance status; TBIL, Total bilirubin; PT, Prothrombin time; ALB, albumin; PLR, platelet to lymphocyte ratio; SIRI, systemic inflammation response index (neutrophil* monocyte to lymphocyte ratio); NLR, neutrophil to lymphocyte ratio; ALB, albumin; AST, aspartate aminotransferase; PVTT, portal vein tumor thrombosis.

### Treatment response and successful conversion surgery

The median follow-up period was 14.4 (10.7–18.2) months. For all patients, the overall response rate was 87.2%, and the disease control rate was 93.6% based on mRECIST (**Table 2**). Among the patients, 32 (34.0%) underwent conversion surgery. The median time to surgery was 3.8 (3.1–5.4) months. All patients who were successfully converted had no distant metastases. Of the entire cohort, 68 (72.3%) patients showed an early tumor response, and 26 (27.7%) had no early tumor response. The patients who showed early tumor response had a significantly higher conversion surgery rate than those who showed no such response (44.1% vs. 7.7%, p=0.001). The patients with early tumor response had similar baseline characteristics to those with no early tumor response, in addition to the ECOG-PS score (p=0.030) and AST (p=0.020) (**Table 1**). Representative cases are presented in **Supplementary Figure 1**.

**Table 2:** Treatment response to conversion therapy based on mRECIST and RECIST1.1.

Overall response	mRECIST (n=94)	RECIST1.1 (n=94)
Complete response	13 (13.8%)	0
Partial response	69 (73.4%)	25 (26.6%)
Stable disease	6 (6.4%)	63 (67.0%)
Progressive disease	6 (6.4%)	6 (6.4%)
Overall response rate	82 (87.2%)	25 (26.6%)
Disease control rate	88 (93.6%)	88 (93.6%)

### Relationship between early tumor response and conversion resection rate

The first multivariate model incorporated early tumor response in the 94 patients included in the study. The result indicates that the only independent predictive factor for conversion surgery was early tumor response (OR=10.296; 95% CI: 2.076–51.063; p=0.004). Early AFP response was included in the second multivariate model; however, early AFP response was not a predictor of conversion resection (**Table 3**). The result was confirmed in 83 patients with non-distant metastases (OR=9.659; 95% CI: 1.899–49.125; p=0.006)(**Table S2**).

**Table 3:** Factors associated with successful conversion surgery in 94 patients who received conversion therapy for initially unresectable HCC.

		Univariate			Multivariate(Model 1)^#^			Multivariate(Model 2)^#^		
		OR	95% CI	*P*	OR	95% CI	*P*	OR	95% CI	*P*
Age, y	> 60 vs ≦ 60	0.662	0.193 – 2.274	0.513						
Sex	Male vs Female	2.138	0.229 – 19.968	0.563						
HBsAg-positive	Yes vs No	1.230	0.296 – 5.117	0.776						
Tumor size, cm	> 10 vs ≦ 10	0.375	0.150 – 0.939	0.036	0.495	0.171 – 1.434	0.195	0.436	0.158– 1.198	0.107
Tumor number	multiple vs single	0.637	0.270 – 1.503	0.303						
PVTT	Yes vs No	0.771	0.328– 1.815	0.552						
Macrovascular invasion	Yes vs No	0.590	0.249 – 1.399	0.231						
BCLC stage	Stage C vs A/B	0.409	0.169 – 0.990	0.047	0.538	0.187– 1.548	0.250	0.414	0.153 – 1.120	0.082
AFP, ng/mL	> 400 vs ≦ 400	0.550	0.231 – 1.308	0.176	0.478	0.169 – 1.347	0.163	0.506	0.191 – 1.344	0.172
SIRI	> 1.38 vs ≦ 1.38	0.378	0.156 – 0.918	0.032	0.459	0.165 – 1.276	0.136	0.454	0.168 – 1.229	0.120
Platelet count,×10^9^/L	> 100 vs≦ 100	2.719	0.304 – 24.325	0.371						
ALT, U/L	> 40 vs ≦ 40	0.992	0.420 – 2.344	0.985						
AST, U/L	> 40 vs ≦ 40	0.642	0.202 – 2.042	0.453						
Ascites	Yes vs No	0.134	0.017 – 1.085	0.060	0.189	0.020 – 1.767	0.144	0.284	0.031 – 2.566	0.262
NLR	> 2.82 vs ≦ 2.82	0.463	0.193 – 1.109	0.084						
PLR	> 146vs ≦ 146	0.463	0.193 – 1.109	0.084						
ALBI grade	Grade1 vs 2/3	0.698	0.172 – 2.836	0.615						
ECOG PS	0 vs 1	1.790	0.740 – 4.329	0.197						
Early AFP response *	Yes vs No	1.548	0.637 – 3.765	0.335	–	–	–	1.803	0.654 – 4.972	0.255
Early tumor response†	Yes vs No	9.474	2.072– 43.309	0.004	**10.296**	**2.076 – 51.063**	**0.004**	–	–	–

AFP, alpha fetoprotein; HCC, hepatocellular carcinoma; ECOG PS, Eastern Cooperative Oncology Group performance status; ALBI grade, albumin-bilirubin grade; ALT, alanine aminotransferase; NA, not adopted; AST, aspartate aminotransferase; BCLC stage, Barcelona-Clinic liver cancer stage; PLR, platelet to lymphocyte ratio; NLR, neutrophil to lymphocyte ratio; SIRI, systemic inflammation response index; CI, confidence interval; OR, odds ratio; PVTT, portal vein tumor thrombosis.

*Early AFP response: AFP reduced > 75% from baseline serum level at first follow-up.

†Early tumor response: Achievement of complete response (CR) and partial response (PR) using mRECIST at first follow-up.

Model 1 did not include early AFP response into multivariate analysis to avoid collinearity.

Model 2 did not include early tumor response into multivariate analysis to avoid collinearity. The bold values denote statistically significant results of the multivariate analysis.

### Effect of early tumor response on PFS and OS

**Figure 2:**
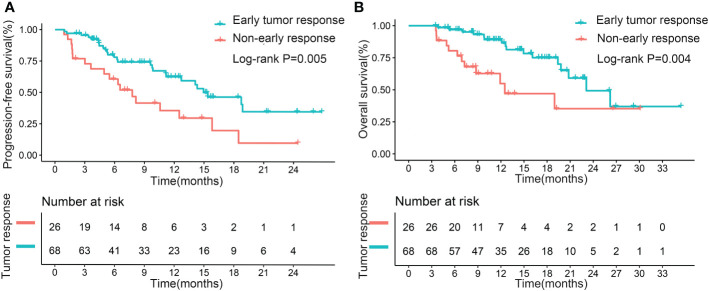
Kaplan–Meier curves for progression-free survival **(A)** and overall survival **(B)**, based on early tumor response in the entire cohort.

The median PFS for the patients with early tumor response was 15.4 months, whereas the median PFS with no early tumor response was 7.8 months (p=0.005; **Figure 2A**). However, early tumor response was not an independent predictive factor of PFS (HR= 0.576, 95% CI: 0.302–1.097; p=0.093) (**Table 4**, Multivariate model 1). The median OS of the patients with early tumor response was 23.1 months (p=0.004; **Figure 2B**), whereas that of patients with no early tumor response was 12.5 months. In multivariate analysis, early tumor response was independently correlated with OS (HR= 0.404, 95% CI: 0.171–0.954; p=0.039) and jointly with the following conditions: baseline AFP>400 ng/mL (p=0.020), portal vein tumor thrombosis (PVTT) (p=0.011), and extrahepatic metastasis (p=0.001)(**Table 5**, Multivariate model 1). The effects of early tumor response on PFS (HR= 0.569, 95% CI: 0.278 – 1.164; p=0.123) and OS (HR= 0.329, 95% CI: 0.119 – 0.910; p=0.032) were confirmed in 83 patients with non-distant metastases (**Tables S3**, **S4**, Multivariate model 1).

**Table 4:** Factors associated with progression-free survival in 94 patients who received conversion therapy for initially unresectable HCC.

		Univariate			Multivariate (Model 1)^#^			Multivariate (Model 2)^#^		
		HR	95% CI	*P*	HR	95% CI	*P*	HR	95%CI	*P*
Age, y	> 60 vs ≦ 60	0.677	0.267 – 1.720	0.412						
Sex	Male vs Female	1.934	0.465 – 8.037	0.364						
HBsAg-positive	Yes vs No	1.725	0.534 – 5.568	0.362						
Tumor size, cm	> 10 vs ≦ 10	1.655	0.837 – 3.274	0.148						
Tumor number	multiple vs single	1.440	0.796 – 2.607	0.228						
PVTT	Yes vs No	1.719	0.939 – 3.146	0.079	1.430	0.745 – 2.746	0.282	1.688	0.851 – 3.348	0.134
Macrovascular invasion	Yes vs No	1.645	0.884 – 3.062	0.116						
Extrahepatic metastasis	Yes vs No	4.117	1.975 – 8.580	< 0.001	**3.583**	**1.659 – 7.738**	**0.001**	**2.439**	**1.111 – 5.356**	**0.026**
BCLC stage	Stage C vs A/B	2.202	1.113 – 4.356	0.023			**NA**			**NA**
BCLC stage	Stage A vs B/C	0.673	0.265 – 1.707	0.404						
AFP, ng/mL	> 400 vs ≦ 400	1.057	0.581 – 1.922	0.857						
SIRI	> 1.38 vs ≦ 1.38	1.751	0.968 – 3.170	0.064						
NLR	> 2.82 vs ≦ 2.82	1.515	0.839 – 2.735	0.168						
PLR	> 146vs ≦ 146	1.049	0.584 – 1.883	0.874						
Platelet count,×10^9^/L	> 100 vs≦ 100	1.575	0.485 – 5.116	0.450						
ALT, U/L	> 40 vs ≦ 40	0.890	0.493 – 1.606	0.699						
AST, U/L	> 40 vs ≦ 40	1.244	0.553 – 2.796	0.597						
Ascites	Yes vs No	2.779	1.318 – 5.860	0.007			**NA**			**NA**
Child-Pugh class	Class A vs B	0.322	0.134 – 0.775	0.011	0.457	0.172 – 1.212	0.115	0.569	0.218 – 1.488	0.251
ALBI grade	Grade1 vs 2/3	1.011	0.399 – 2.566	0.981						
ECOG PS	0 vs 1	0.525	0.290 – 0.950	0.033	0.770	0.399 – 1.487	0.436	0.724	0.373 – 1.404	0.339
Early AFP response *	Yes vs No	0.378	0.181– 0.789	0.010	–	–	–	0.585	0.270 – 1.268	0.175
Early tumor response†	Yes vs No	0.433	0.237 – 0.789	0.006	0.576	0.302 – 1.097	0.093	–	–	–
Successful conversion surgery	Yes vs No	0.197	0.086 –0.454	<0.001	–	–	–	**0.248**	**0.099– 0.622**	**0.003**

AFP, alpha fetoprotein; HCC, hepatocellular carcinoma; ECOG PS, Eastern Cooperative Oncology Group performance status; ALBI grade, albumin-bilirubin grade; ALT, alanine aminotransferase; NA, not adopted; AST, aspartate aminotransferase; BCLC stage, Barcelona-Clinic liver cancer stage; PLR, platelet to lymphocyte ratio; NLR, neutrophil to lymphocyte ratio; SIRI, systemic inflammation response index; CI, confidence interval; OR, odds ratio; PVTT, portal vein tumor thrombosis.

*Early AFP response: AFP reduced > 75% from baseline serum level at first follow-up.

†Early tumor response: Achievement of complete response (CR) and partial response (PR) using mRECIST at first follow-up.

Model 1 did not include early AFP response, successful conversion surgery, BCLC stage, and ascites into multivariate analysis to avoid collinearity.

Model 2 did not include early tumor response, BCLC stage, and ascites into multivariate analysis to avoid collinearity. The bold values denote statistically significant results of the multivariate analysis.

**Table 5:** Factors associated with overall survival in 94 patients who received conversion therapy for initially unresectable HCC.

		Univariate			Multivariate (Model 1)^#^			Multivariate (Model 2)^#^		
		HR	95% CI	*P*	HR	95% CI	*P*	HR	95%CI	*P*
Age, y	> 60 vs ≦ 60	1.162	0.399 – 3.383	0.660						
Sex	Male vs Female	0.990	0.233 – 4.201	0.989						
HBsAg-positive	Yes vs No	3.011	0.408 – 22.202	0.280						
Tumor size, cm	> 10 vs ≦ 10	1.314	0.555 – 3.107	0.535						
Tumor number	multiple vs single	1.375	0.646 – 2.924	0.408						
PVTT	Yes vs No	2.551	1.121 – 5.806	0.026	**3.014**	**1.290 – 7.042**	**0.011**	**4.418**	**1.712 – 11.398**	**0.002**
Macrovascular invasion	Yes vs No	2.410	1.020 – 5.691	0.045			**NA**			**NA**
Extrahepatic metastasis	Yes vs No	4.651	1.918 – 11.275	0.001	**5.068**	**1.897 – 13.534**	**0.001**	**3.189**	**1.172 – 8.678**	**0.023**
BCLC stage	Stage C vs A/B	3.084	1.168 – 8.139	0.023			**NA**			**NA**
BCLC stage	Stage A vs B/C	0.571	0.172 – 1.899	0.361						
AFP, ng/mL	> 400 vs ≦ 400	2.024	0.890 – 4.601	0.092	**3.002**	**1.190 – 7.575**	**0.020**	2.468	0.957 – 6.367	0.062
SIRI	> 1.38 vs ≦ 1.38	2.172	1.006 – 4.688	0.048	2.144	0.900 – 5.110	0.085	2.098	0.844 – 5.214	0.111
NLR	> 2.82 vs ≦ 2.82	1.688	0.796 – 3.580	0.172						
PLR	> 146vs ≦ 146	1.368	0.648 – 2.886	0.411						
Platelet count,×10^9^/L	> 100 vs≦ 100	2.990	0.405 – 22.080	0.283						
ALT, U/L	> 40 vs ≦ 40	1.123	0.531 – 2.378	0.761						
AST, U/L	> 40 vs ≦ 40	1.015	0.382 – 2.698	0.977						
Ascites	Yes vs No	2.232	0.831 – 5.998	0.111						
Child-Pugh class	Class A vs B	0.245	0.082 – 0.735	0.012	0.754	0.209 –2.711	0.665	0.880	0.255 – 3.036	0.840
ALBI grade	Grade1 vs 2/3	1.437	0.497 – 4.154	0.503						
ECOG PS	0 vs 1	0.577	0.269 – 1.238	0.158	0.707	0.300 – 1.669	0.429	0.680	0.281 – 1.645	0.392
Early AFP response *	Yes vs No	0.445	0.180– 1.102	0.080	–	–	–	0.580	0.210 – 1.600	0.293
Early tumor response†	Yes vs No	0.348	0.163 – 0.743	0.006	**0.404**	**0.171 – 0.954**	**0.039**	–	–	–
Successful conversion surgery	Yes vs No	0.135	0.044 –0.409	<0.001	–	–	–	**0.147**	**0.039 – 0.554**	**0.005**

AFP, alpha fetoprotein; HCC, hepatocellular carcinoma; ECOG PS, Eastern Cooperative Oncology Group performance status; ALBI grade, albumin-bilirubin grade; ALT, alanine aminotransferase; NA, not adopted; AST, aspartate aminotransferase; BCLC stage, Barcelona-Clinic liver cancer stage; PLR, platelet to lymphocyte ratio; NLR, neutrophil to lymphocyte ratio; SIRI, systemic inflammation response index; CI, confidence interval; OR, odds ratio; PVTT, portal vein tumor thrombosis.

* Early AFP response: AFP reduced > 75% from baseline serum level at first follow-up.

† Early tumor response: Achievement of complete response (CR) and partial response (PR) using mRECIST at first follow-up.

Model 1 did not include successful conversion surgery, early AFP response, macrovascular invasion, and BCLC stage into multivariate analysis to avoid collinearity.

Model 2 did not include early tumor response, macrovascular invasion, and BCLC stage into multivariate analysis to avoid collinearity. The bold values denote statistically significant results of the multivariate analysis.

**Figure 3:**
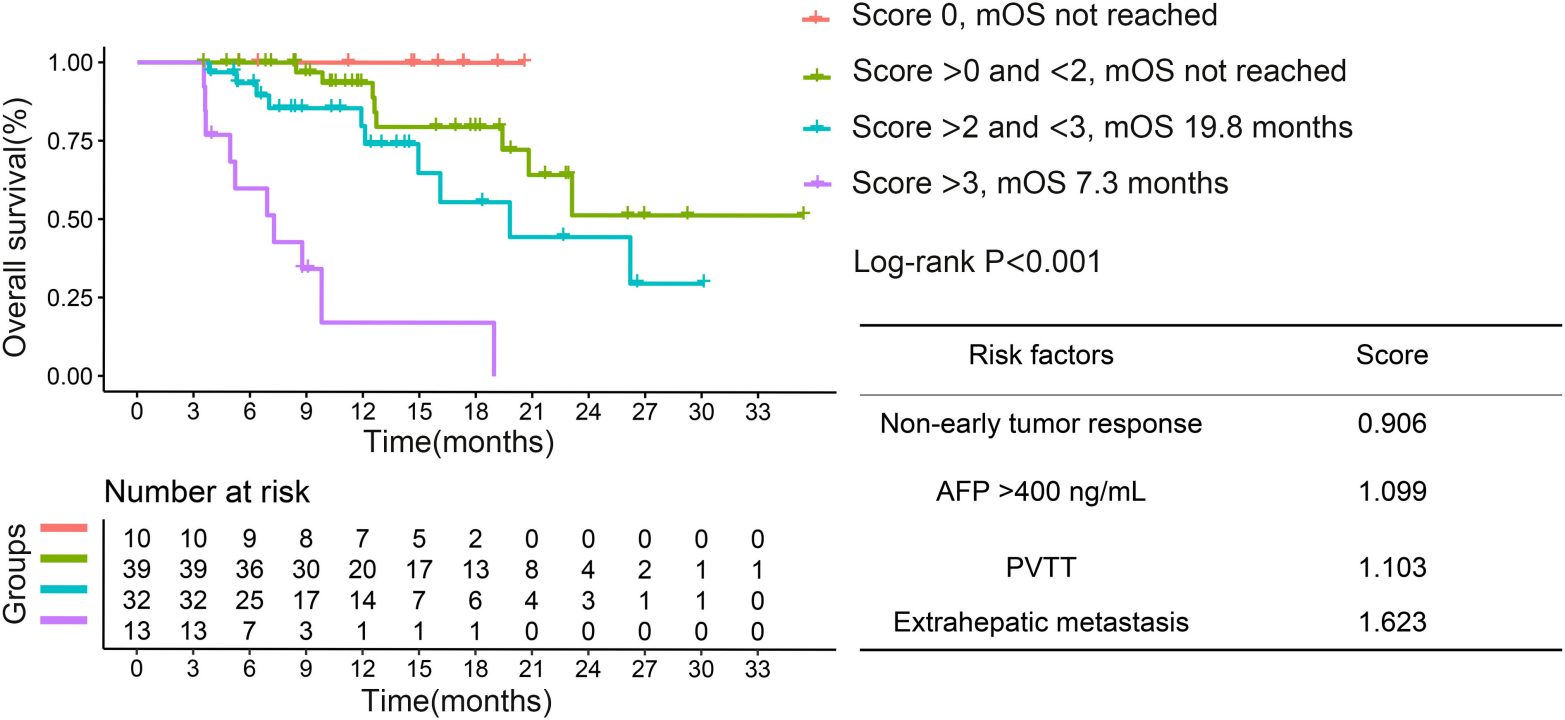
Kaplan–Meier curves for overall survival based on different risk factor scores in the entire cohort. PVTT, portal vein tumor thrombosis; AFP, α-fetoprotein; OS, overall survival.

Given that non-early tumor response was a risk factor for OS, we assigned different scores to each risk factor on the basis of the beta coefficient score in multivariate analysis. We further divided the patients into 4 groups, based on the risk factor. Survival curves showed that the best median OS was in the score 0 group, which declined as the risk factor scores increased (**Figure 3**).

### Relevance of successful conversion surgery for PFS and OS

**Figure 4:**
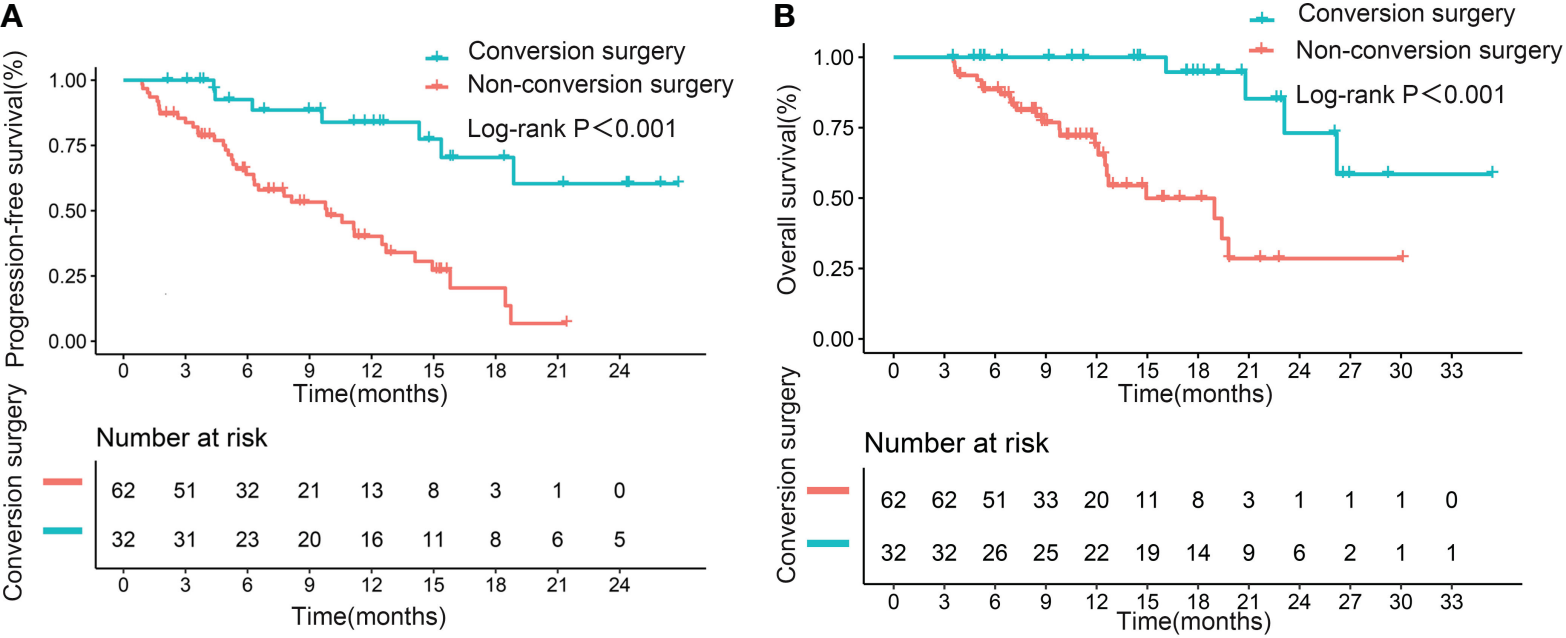
Kaplan–Meier curves for progression-free survival **(A)** and overall survival **(B)**, based on successful conversion surgery, in the entire cohort.

**Figure 5:**
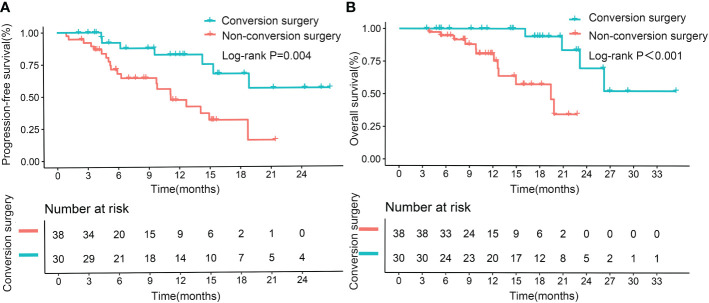
Kaplan–Meier curves for progression-free survival **(A)** and overall survival **(B)** in patients with early tumor response, based on successful conversion surgery.

Median PFS and OS (not reached, not reached) were significantly longer in patients who underwent successful conversion surgery than in those patients who received no such surgery (9.8 months, p<0.001; 14.9 months, p<0.001; **Figures 4A, B**). We further assessed the role of conversion surgery in patients with early tumor response. Results showed that for patients with early tumor response, those who underwent conversion surgery also had significantly longer median PFS and OS (not reached, not reached) than those who did not undergo conversion resection (11.2 months, p=0.004; 19.4 months, p<0.001; **Figures 5A, B**). Furthermore, early responders combined with late responders had a similar median OS to that of early responders (p=1.000). Both had significantly longer OS than non-responders (p<0.001, p<0.001; respectively; **Supplementary Figure 2A**). We further assessed the role of conversion surgery in late tumor responders. The results showed that late responders who underwent conversion surgery had no significantly longer median OS than those who did not (p=0.3; **Supplementary Figure 2B**).

Multivariate results confirmed that successful conversion surgery (HR=0.248, 95% CI: 0.099–0.622; p=0.003) was independently correlated with PFS (**Table 4**, Multivariate model 2). In addition, multivariate analysis confirmed that successful conversion surgery was also independently correlated with OS (HR = 0.147, 95% CI: 0.039 – 0.554; p=0.005), in addition to PVTT (p=0.002) and extrahepatic metastasis (p=0.023)(**Table 5**, Multivariate model 2). The results on the relevance of successful conversion surgery for PFS (HR= 0.265, 95% CI: 0.105– 0.669; p=0.005) and OS (HR= 0.088, 95% CI: 0.021 – 0.363; p=0.001) were confirmed in 83 patients with non-distant metastases (**Table S3**, **S4**, Multivariate model 2).

## Discussion

The triple-combination LTP is a trend in conversion therapy for patients with iuHCC (5). However, LTP provides improved treatment outcomes while challenging the prediction of successful conversion surgery and prognosis. We found that early tumor response was independently associated with successful conversion surgery and better survival. Moreover, successful conversion surgery after LTP is essential for a better prognosis, especially early responders.

Some markers might help predict the prognosis of people with liver cancer. NLR is a prognostic factor in iuHCC for patients receiving triple-combination therapy (20). Baseline PLR and SIRI are also associated with the prognosis for HCC (9, 25, 26). In addition, the combination of C-reactive protein (CRP) and AFP showed a prognostic role in HCC patients receiving tyrosine kinase inhibitors (TKIs) combined with immunotherapy (27, 28). However, CRP is not a mandatory test for every patient in our hospital, hence its limited application in current clinical practice. To explore their value in conversion therapy, we chose inflammatory indexes in blood routine, such as PLR, NLR, and SIRI substituted for CRP. Further, we used the baseline AFP and early AFP response to explore the potential predictive factor. However, among the aforementioned indicators, only baseline AFP was independently associated with OS. Therefore, the above indicators have a limited role in LTP conversion therapy.

Several studies have been conducted on the prognostic role of early tumor response in iuHCC, but the results have been inconclusive. Earlier studies by Hashi et al. and Öcal et al. evaluated the relationship between survival and early tumor response in patients treated with TKI, in accordance with RECIST and mRECIST, respectively (18, 19). They found that early tumor response was independently associated with prognosis. Current studies recommend mRECIST-based tumor response assessment, given that RECIST is not considered applicable for viable tumors (18, 29, 30). However, a real-world study indicated that early tumor response based on mRECIST was not correlated with prognosis in patients receiving PD-1 inhibitors combined with bevacizumab (21). The study found that the Choi criteria and revised Choi criteria, which consider tumor density on CE-CT, might provide a more suitable evaluation of early tumor response and its correlation with prognosis (21). We considered that applying the Choi criteria and revised Chio criteria is not preferable because of the use of TACE in triple-combination therapy. Therefore, we still chose mRECIST to evaluate early tumor response. We determined that early tumor response was the only factor independently correlated with successful conversion resection. The result is also applicable to patients without distant metastases. A significant association was also found between early tumor response and OS but not between early tumor response and PFS, a finding that is inconsistent with previous results (20). The main reason is that in the previous study, only two patients underwent conversion surgery, whereas in the current study, 34.0% of the patients underwent conversion resection. Therefore, early tumor response is an important impact factor in conversion surgery and the prognosis of patients undergoing LTP conversion therapy. We further included early tumor response for risk stratification and found that OS decreased with increasing risk factor scores. It demonstrates the potential clinical value of this stratification that takes into account early tumor response and provides evidence to support the importance of future larger-scale studies.

Conversion resection may be the only means to obtain a cure for patients with iuHCC (2). No more evidence of whether surgical resection should be performed after conversion therapy for the treatment of iuHCC meets the criteria for resectability. In our multivariable prognosis analysis, conversion surgery was an independent predictor of prognosis. This finding suggests that conversion surgery is important and necessary. To further identify the significance of conversion surgery, we compared the survival of patients with early tumor response who received conversion surgery with the survival of patients who were yet to meet the criteria for resectability despite a favorable early tumor response. We also found a better prognosis for patients who had undergone conversion surgery. Therefore, hepatectomy should be performed if patients meet the criteria for resection in early responders. However, late responders only demonstrated a trend toward prolonged OS after conversion surgery, with no significant difference in OS. In addition, late responders had low conversion surgery rate and may be no less effective than early responders. Further sample size expansion is required to confirm this result.Therefore, conversion surgery significantly improved the prognosis in the overall population, but its efficacy in late responders requires further testing.

Several limitations should be noted. First, the small sample size in one hospital prevented us from further exploring the factors influencing the prognosis of patients who received successful conversion surgery. Second, potential selection bias is inevitable because of the retrospective nature of the study. A large sample of prospective studies is necessary to further explore the subject. Third, we used two PD-1 inhibitors. Although no differences in the prognostic impact of these immunotherapeutic drugs were found (data not shown), further studies on the effects of different PD-1 inhibitors on conversion therapy are still needed.

In summary, early tumor response is an important predictive marker for successful conversion surgery and prolonged survival in patients with iuHCC treated using LTP conversion therapy. Conversion surgery is necessary to improve survival during conversion therapy, particularly for early responders.

## Data availability statement

The raw data supporting the conclusions of this article will be made available by the authors, without undue reservation.

## Ethics statement

The studies involving human participants were reviewed and approved by Ethics Committee of Guangxi Medical University Cancer Hospital(approval number: LW2022147). The patients/participants provided their written informed consent to participate in this study. Written informed consent was obtained from the individual(s) for the publication of any potentially identifiable images or data included in this article.

## Author contributions

XL, JC, LL and FW contributed for study concept, design, and finished manuscript writing. XL, JC, XW, TB, and SL performed data analysis and critically revised the article. XL, JC, TW, ZT, CH, BZ, and BL were involved in data collection and verification. All authors contributed to the article and approved the final manuscript.
