# Supplementary material for: Outcomes and prognostic factors in initially unresectable hepatocellular carcinoma treated using conversion therapy with lenvatinib and TACE plus PD-1 inhibitors
Source: Front Oncol. 2023 Jan 30;13:1110689. doi: 10.3389/fonc.2023.1110689 (PMC9923097; doi:10.3389/fonc.2023.1110689)
Supplement: Supplementary file 1 [file Table_1.docx]

**Supplementary material**

**Procedure of TACE**

TACE included conventional TACE (C-TACE) and drug-eluting beads TACE (d-tacE). With the Seldinger technique, a 4F-to-5F French catheter was introduced into the abdominal aorta under local anesthesia. Hepatic arterial angiography was performed using fluoroscopy to guide the catheter into the celiac and superior mesenteric arteries. Subsequently, the feeding arteries, tumor stains, and vascular anatomy surrounding the tumor were identified. A microcatheter was then introduced via the catheter to reach the feeding arteries. For patients undergoing C-TACE, an emulsion of 5–15 mL lipiodol (Andre Guerbet, Aulnay-sous-Bois, France), as a drug carrier, was mixed with chemotherapy drugs. In addition, microspheres (8spheres^®^, Heng Rui Callisyn Biomedical, 20153131072) with different diameters, as embolic agents, were injected into tumors. Patients receiving D-TACE were embolized with chemotherapeutic drugs loaded with drug-eluting beads (Callispheres^®^, Heng Rui Callisyn Biomedical, 20153131072). The treatment was deemed completed once the blood flow almost stopped. Chemotherapy drugs and doses varied depending on individual tumor status.

**Table S1. Conversion therapy-related adverse events.**

| Adverse event | Any grade | Grade 3/4 |
| --- | --- | --- |
| ALT increased | 86(91.5%) | 29(30.9%) |
| AST increased | 93(98.9%) | 54(57.4%) |
| Albumin decreased | 29(30.9%) | 0 |
| Total bilirubin increased | 53(56.4%) | 2(2.1%) |
| Hypertension | 12(12.8%) | 4(4.3%) |
| Pain | 55(58.5%) | 12(12.8%) |
| Fever | 12(12.8%) | 0 |
| Proteinuria | 6(6.4%) | 0 |
| Fatigue | 5(5.3%) | 0 |
| Hand-foot syndrome | 19(20.2%) | 2(2.1%) |
| Skin and subcutaneous tissue diseases | 7(7.4%) | 0 |
| Diarrhea | 5(5.3%) | 0 |
| Nausea | 18(19.4%) | 0 |
| Decreased appetite | 5(5.3%) | 1(1.1%) |
| Weight drop | 8(8.5%) | 0 |
| Edema peripheral | 2(2.1%) | 0 |
| Decreased platelet count | 7(7.4%) | 1(1.1%) |
| Decreased white blood cell | 7(7.4%) | 1(1.1%) |

Abbreviations: AST, aspartate aminotransferase; ALT, alanine aminotransferase.

**Table S2. Factors associated with successful conversion surgery in 83 patients who received conversion therapy for initially unresectable HCC without distant metastasis.**

|  |  | Univariate | | |  | Multivariate(Model 1)^#^ | | | Multivariate(Model 2)^#^ | | |
| --- | --- | --- | --- | --- | --- | --- | --- | --- | --- | --- | --- |
|  |  | OR | 95% CI | *P* |  | OR | 95% CI | *P* | OR | 95% CI | *P* |
| Age, y | > 60 vs ≦ 60 | 0.898 | 0.241 – 3.350 | 0.873 |  |  |  |  |  |  |  |
| Sex | Male vs Female | 1.937 | 0.193 – 19.476 | 0.574 |  |  |  |  |  |  |  |
| HBsAg-positive | Yes vs No | 1.051 | 0.233 – 4.732 | 0.949 |  |  |  |  |  |  |  |
| Tumor size, cm | > 10 vs ≦ 10 | 0.440 | 0.172 – 1.126 | 0.087 |  | 0.536 | 0.182 – 1.576 | 0.257 | 0.511 | 0.184 – 1.420 | 0.198 |
| Tumor number | multiple vs single | 0.669 | 0.275 – 1.627 | 0.376 |  |  |  |  |  |  |  |
| PVTT | Yes vs No | 0.759 | 0.312 – 1.842 | 0.542 |  |  |  |  |  |  |  |
| Macrovascular  invasion | Yes vs No | 0.545 | 0.222– 1.342 | 0.187 |  |  |  |  |  |  |  |
| BCLC stage | Stage C vs A/B | 0.545 | 0.222 – 1.342 | 0.187 |  | 0.679 | 0.233 – 1.980 | 0.478 | 0.555 | 0.203 – 1.517 | 0.251 |
| AFP, ng/mL | > 400 vs ≦ 400 | 0.457 | 0.184 – 1.137 | 0.092 |  | 0.379 | 0.132 – 1.090 | 0.072 | 0.392 | 0.144 – 1.065 | 0.066 |
| SIRI | > 1.38 vs ≦ 1.38 | 0.430 | 0.172 – 1.074 | 0.071 |  |  |  |  |  |  |  |
| NLR | > 2.82 vs ≦ 2.82 | 0.493 | 0.200 – 1.217 | 0.125 |  |  |  |  |  |  |  |
| PLR | > 146vs ≦ 146 | 0.455 | 0.184 – 1.125 | 0.088 |  |  |  |  |  |  |  |
| Platelet count,×10^9^/L | > 100 vs≦ 100 | 2.638 | 0.282 – 24.726 | 0.395 |  |  |  |  |  |  |  |
| ALT, U/L | > 40 vs ≦ 40 | 0.900 | 0.368 – 2.200 | 0.817 |  |  |  |  |  |  |  |
| AST, U/L | > 40 vs ≦ 40 | 0.369 | 0.095 – 1.427 | 0.148 |  |  |  |  |  |  |  |
| Ascites | Yes vs No | 1.132 | 0.016 – 1.089 | 0.060 |  | 0.127 | 0.014 – 1.192 | 0.071 | 0.172 | 0.019 – 1.573 | 0.119 |
| ALBI grade | Grade1vs 2/3 | 0.556 | 0.136 – 2.273 | 0.414 |  |  |  |  |  |  |  |
| ECOG PS | 0 vs 1 | 1.697 | 0.681 – 4.231 | 0.257 |  |  |  |  |  |  |  |
| Early AFP response * | Yes vs No | 1.368 | 0.548 – 3.416 | 0.502 |  | – | – | – | 1.656 | 0.602 – 4.560 | 0.329 |
| Early tumor response† | Yes vs No | 8.182 | 1.750– 38.252 | 0.008 |  | **9.659** | **1.899 – 49.125** | **0.006** | – | – | – |

Abbreviations:

AFP, alpha fetoprotein; HCC, hepatocellular carcinoma; ECOG PS, Eastern Cooperative Oncology Group performance status; ALBI grade, albumin-bilirubin grade; ALT, alanine aminotransferase; NA, not adopted; AST, aspartate aminotransferase; BCLC stage, Barcelona-Clinic liver cancer stage; PLR, platelet to lymphocyte ratio; NLR, neutrophil to lymphocyte ratio; SIRI, systemic inﬂammation response index; CI, confidence interval; OR, odds ratio; PVTT, portal vein tumor thrombosis.
* Early AFP response : AFP reduced > 75% from baseline serum level at first follow-up.

† Early tumor response: Achievement of complete response (CR) and partial response (PR) using mRECIST at first follow-up.

^#^ Model 1 did not include early AFP response into multivariate analysis to avoid collinearity.

^#^ Model 2 did not include early tumor response into multivariate analysis to avoid collinearity.

**Table S3. Factors associated with progression-free survival in 83 patients who received conversion therapy for initially unresectable HCC without distant metastasis.**

|  |  | Univariate | | |  | Multivariate (Model 1)^#^ | | |  |  | Multivariate (Model 2)^#^ | | |
| --- | --- | --- | --- | --- | --- | --- | --- | --- | --- | --- | --- | --- | --- |
|  |  | HR | 95% CI | *P* |  | HR | 95% CI | *P* |  |  | HR | 95%CI | *P* |
| Age, y | > 60 vs ≦ 60 | 0.383 | 0.092 – 1.597 | 0.188 |  |  |  |  |  |  |  |  |  |
| Sex | Male vs Female | 1.460 | 0.347 – 6.153 | 0.606 |  |  |  |  |  |  |  |  |  |
| HBsAg-positive | Yes vs No | 1.855 | 0.445 – 7.739 | 0.396 |  |  |  |  |  |  |  |  |  |
| Tumor size, cm | > 10 vs ≦ 10 | 1.431 | 0.685 – 2.988 | 0.340 |  |  |  |  |  |  |  |  |  |
| Tumor number | multiple vs single | 1.465 | 0.748 – 2.868 | 0.265 |  |  |  |  |  |  |  |  |  |
| PVTT | Yes vs No | 1.756 | 0.883 – 3.492 | 0.109 |  | 1.292 | 0.592 – 2.819 | 0.520 |  |  | 1.381 | 0.600 – 3.178 | 0.448 |
| Macrovascular  invasion | Yes vs No | 1.755 | 0.858 – 3.590 | 0.123 |  |  |  |  |  |  |  |  |  |
| BCLC stage | Stage C vs A/B | 1.755 | 0.858 – 3.590 | 0.123 |  |  |  | **NA** |  |  |  |  | **NA** |
| AFP, ng/mL | > 400 vs ≦ 400 | 1.123 | 0.565 – 2.233 | 0.741 |  |  |  |  |  |  |  |  |  |
| SIRI | > 1.38 vs ≦ 1.38 | 1.651 | 0.846 – 3.220 | 0.141 |  |  |  |  |  |  |  |  |  |
| NLR | > 2.82 vs ≦ 2.82 | 1.591 | 0.815 – 3.104 | 0.174 |  |  |  |  |  |  |  |  |  |
| PLR | > 146vs ≦ 146 | 1.037 | 0.534 – 2.014 | 0.915 |  |  |  |  |  |  |  |  |  |
| Platelet count,×10^9^/L | > 100 vs≦ 100 | 1.830 | 0.435 – 7.701 | 0.410 |  |  |  |  |  |  |  |  |  |
| ALT, U/L | > 40 vs ≦ 40 | 0.997 | 0.507 – 1.961 | 0.993 |  |  |  |  |  |  |  |  |  |
| AST, U/L | > 40 vs ≦ 40 | 1.984 | 0.606 – 6.498 | 0.258 |  |  |  |  |  |  |  |  |  |
| Child-Pugh class | Class A vs B | 0.379 | 0.132 – 1.093 | 0.073 |  | 0.506 | 0.163 – 1.573 | 0.239 |  | 0. | 0.734 | 0.233 – 2.312 | 0.597 |
| ALBI grade | Grade1 vs 2/3 | 1.256 | 0.486 – 3.244 | 0.638 |  |  |  |  |  |  |  |  |  |
| ECOG PS | 0 vs 1 | 0.477 | 0.243 – 0.935 | 0.031 |  | 0.594 | 0.279 – 1.265 | 0.177 |  |  | 0.481 | 0.224 – 1.032 | 0.060 |
| Early AFP response * | Yes vs No | 0.346 | 0.150– 0.798 | 0.013 |  | – | – | – |  |  | 0.445 | 0.180 – 1.101 | 0.080 |
| Early tumor response† | Yes vs No | 0.460 | 0.231 – 0.917 | 0.027 |  | 0.569 | 0.278 – 1.164 | 0.123 |  |  | – | – | – |
| Successful conversion surgery | Yes vs No | 0.232 | 0.099 –0.547 | 0.001 |  | – | – | – |  |  | **0.265** | **0.105– 0.669** | **0.005** |

Abbreviations:

AFP, alpha fetoprotein; HCC, hepatocellular carcinoma; ECOG PS, Eastern Cooperative Oncology Group performance status; ALBI grade, albumin-bilirubin grade; ALT, alanine aminotransferase; NA, not adopted; AST, aspartate aminotransferase; BCLC stage, Barcelona-Clinic liver cancer stage; PLR, platelet to lymphocyte ratio; NLR, neutrophil to lymphocyte ratio; SIRI, systemic inﬂammation response index; CI, confidence interval; OR, odds ratio; PVTT, portal vein tumor thrombosis.
* Early AFP response : AFP reduced > 75% from baseline serum level at first follow-up.

† Early tumor response: Achievement of complete response (CR) and partial response (PR) using mRECIST at first follow-up.

^#^ Model 1 did not include early AFP response, successful conversion surgery, and BCLC stage into multivariate analysis to avoid collinearity.

^#^ Model 2 did not include early tumor response and BCLC stage into multivariate analysis to avoid collinearity.

**Table S4. Factors associated with overall survival in 83 patients who received conversion therapy for initially unresectable HCC without distant metastasis.**

|  |  | Univariate | | |  | Multivariate (Model 1)^#^ | | |  |  | Multivariate (Model 2)^#^ | | | | |
| --- | --- | --- | --- | --- | --- | --- | --- | --- | --- | --- | --- | --- | --- | --- | --- |
|  |  | HR | 95% CI | *P* |  | HR | 95% CI | *P* | | |  |  | HR | 95%CI | *P* |
| Age, y | > 60 vs ≦ 60 | 0.875 | 0.202– 3.787 | 0.858 |  |  |  |  | | |  |  |  |  |  |
| Sex | Male vs Female | 0.733 | 0.168 – 3.188 | 0.678 |  |  |  |  | | |  |  |  |  |  |
| HBsAg-positive | Yes vs No | 2.055 | 0.275 – 15.360 | 0.483 |  |  |  |  | | |  |  |  |  |  |
| Tumor size, cm | > 10 vs ≦ 10 | 1.133 | 0.436 – 2.945 | 0.798 |  |  |  |  | | |  |  |  |  |  |
| Tumor number | multiple vs single | 1.487 | 0.621 – 3.558 | 0.373 |  |  |  |  | | |  |  |  |  |  |
| PVTT | Yes vs No | 2.457 | 0.950 – 6.352 | 0.064 |  | **3.091** | **1.073 – 8.907** | **0.037** | | |  |  | **5.231** | **1.737 – 15.749** | **0.003** |
| Macrovascular  invasion | Yes vs No | 2.404 | 0.876 – 6.600 | 0.089 |  |  |  | **NA** | | |  |  |  |  | **NA** |
| BCLC stage | Stage C vs A/B | 2.404 | 0.876 – 6.600 | 0.089 |  |  |  | **NA** | | |  |  |  |  | **NA** |
| AFP, ng/mL | > 400 vs ≦ 400 | 2.421 | 0.886 – 6.618 | 0.085 |  | **4.800** | **1.448– 15.919** | **0.010** | | |  |  | 2.866 | 0.844 – 9.737 | 0.091 |
| SIRI | > 1.38 vs ≦ 1.38 | 2.237 | 0.931 – 5.376 | 0.072 |  | 2.431 | 0.935 – 6.322 | 0.069 | | |  |  | 1.964 | 0.748 – 5.156 | 0.170 |
| NLR | > 2.82 vs ≦ 2.82 | 1.977 | 0.831 – 4.704 | 0.123 |  |  |  |  | | |  |  |  |  |  |
| PLR | > 146vs ≦ 146 | 1.083 | 0.458 – 2.562 | 0.856 |  |  |  |  | | |  |  |  |  |  |
| ALT, U/L | > 40 vs ≦ 40 | 1.274 | 0.527 – 3.080 | 0.591 |  |  |  |  | | |  |  |  |  |  |
| AST, U/L | > 40 vs ≦ 40 | 1.006 | 0.292 – 3.463 | 0.992 |  |  |  |  | | |  |  |  |  |  |
| Ascites | Yes vs No | 2.761 | 0.904 – 8.434 | 0.075 |  |  |  |  | | |  |  |  |  |  |
| Child-Pugh class | Class A vs B | 0.354 | 0.079 – 1.582 | 0.174 |  | 1.625 | 0.276 –9.557 | 0.591 | | |  | 0. | 1.923 | 0.378 – 9.790 | 0.431 |
| ALBI grade | Grade1 vs 2/3 | 1.884 | 0.632 – 5.620 | 0.256 |  |  |  |  | | |  |  |  |  |  |
| ECOG PS | 0 vs 1 | 0.456 | 0.187 – 1.110 | 0.084 |  | 0.447 | 0.154 – 1.295 | 0.138 | | |  |  | 0.420 | 0.140 – 1.258 | 0.121 |
| Early AFP response * | Yes vs No | 0.345 | 0.116– 1.028 | 0.056 |  |  |  |  | | |  |  |  |  |  |
| Early tumor response† | Yes vs No | 0.400 | 0.164– 0.975 | 0.044 |  | **0.329** | **0.119 – 0.910** | **0.032** | | |  |  | – | – | – |
| Successful conversion surgery | Yes vs No | 0.144 | 0.046 –0.452 | 0.001 |  | – | – | – | | |  |  | **0.088** | **0.021 – 0.363** | **0.001** |

Abbreviations:

AFP, alpha fetoprotein; HCC, hepatocellular carcinoma; ECOG PS, Eastern Cooperative Oncology Group performance status; ALBI grade, albumin-bilirubin grade; ALT, alanine aminotransferase; NA, not adopted; AST, aspartate aminotransferase; BCLC stage, Barcelona-Clinic liver cancer stage; PLR, platelet to lymphocyte ratio; NLR, neutrophil to lymphocyte ratio; SIRI, systemic inﬂammation response index; CI, confidence interval; OR, odds ratio; PVTT, portal vein tumor thrombosis.
* Early AFP response : AFP reduced > 75% from baseline serum level at first follow-up.

† Early tumor response: Achievement of complete response (CR) and partial response (PR) using mRECIST at first follow-up.

^#^ Model 1 did not include successful conversion surgery, macrovascular invasion, and BCLC stage into multivariate analysis to avoid collinearity.

^#^ Model 2 did not include early tumor response, macrovascular invasion, and BCLC stage into multivariate analysis to avoid collinearity.

**
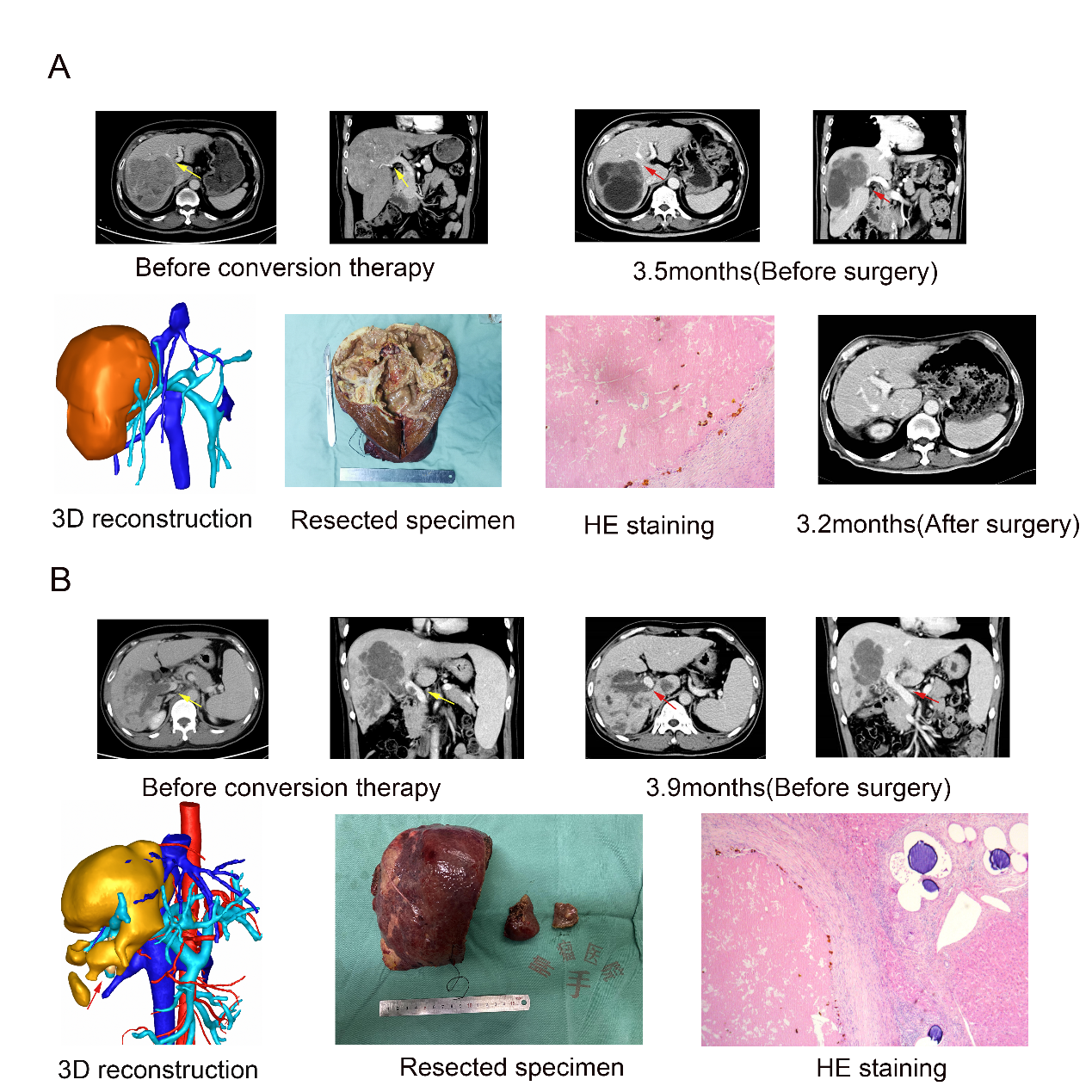
**

**Supplemental Figure 1.** Two representative cases. (A) A patient was diagnosed with right hepatic lobe HCC (BCLC-A). However, the tumor did not meet the R0 resection criteria because of the large tumor burden, and the tumor was close to middle hepatic vein **(yellow arrows)**. After conversion therapy for 3.5 months, the tumor shrinkage was observed on enhanced CT and 3D reconstruction images, meeting the R0 resection criteria **(red arrows)**. Curative right hemihepatectomy was thus performed. Hematoxylin–eosin (H&E) staining of the surgically resected specimen showed major pathologic response. At 3.2 months after surgery, enhanced CT showed no abnormal enhancement in the residual liver. (B) A patient was diagnosed with right hepatic lobe HCC invading the right branch and trunk of the portal vein **(yellow arrows)**(BCLC-C). After conversion therapy for 3.9 months, regression of the cancer thrombus with the restoration of portal venous blood flow was observed on enhanced CT and 3D reconstruction images **(red arrows)**. Curative right hemihepatectomy was performed, and H&E staining of the surgically resected specimen showed only a small number of surviving tumor cells. BCLC, Barcelona clinical liver cancer ;3D; three-dimensional.


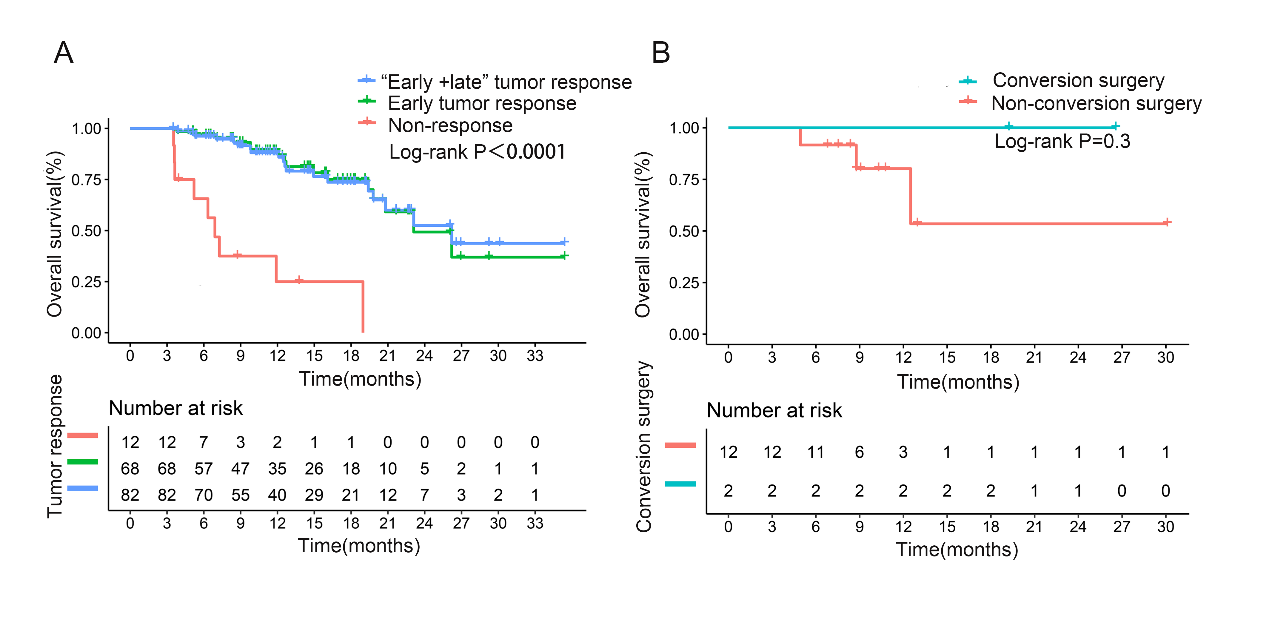


**Supplemental Figure 2.** Kaplan–Meier curves for overall survival in patients with different tumor response (A); Kaplan–Meier curves for overall survival in patients with late tumor response, based on successful conversion surgery (B).
